# Derivation and validation of an accurate estimation of CD4 counts from the absolute lymphocyte count in virologically suppressed and immunologically reconstituted HIV infected adults

**DOI:** 10.1186/s12879-015-1079-5

**Published:** 2015-08-13

**Authors:** Barnaby Young, Oon Tek Ng, David Chien Lye, Yee Sin Leo

**Affiliations:** Institute of Infectious Diseases and Epidemiology, , Tan Tock Seng Hospital, Singapore, Singapore; Saw Swee Hock School of Public Health, National University of Singapore, Singapore, Singapore; Yong Yoo Lin School of Medicine, National University of Singapore, Singapore, Singapore

## Abstract

**Background:**

A simple method to estimate CD4 counts in stable, HIV infected virologically-suppressed and immune-reconstituted adults could save the expense of unnecessary formal testing.

**Methods:**

Using a baseline CD4 percent, CD4 counts were estimated from subsequent absolute lymphocyte counts (ALC) measured by an automated FBC machine (CD4 estimate calculated by the ALC multiplied by the baseline CD4 percent). The accuracy of this approach was established in a large, retrospective clinical laboratory dataset of virologically-suppressed HIV infected subjects. A case–control study explored important clinical factors for accurate estimates, and a heuristic algorithm was derived and validated in a random sample.

**Results:**

Data from 3,630 subjects were available. CD4 counts were generally accurately estimated, with a mean 6.1 % underestimation. Overall 83.3 % of CD4 estimates were within 25 % of the actual values, with 12.1 % CD4 counts underestimated by more than 25 %, and 4.5 % overestimated. The CD4 count was increasingly underestimated with time from baseline, and the degree of underestimation correlated with baseline CD4 percent (p < 0.0001). From the case–control study, baseline CD4 percent of ≥20, no illness requiring hospitalization and more than a year since starting or switch of anti-retroviral therapy were identified as significant predictors of inaccurate estimates. Employing this simple algorithm, CD4 estimate accuracy improved to a mean 1.3 % underestimation, and the proportion of estimates within 25 % of the actual value increased to 93.4 %.

**Conclusions:**

In virologically-suppressed and immune-reconstituted HIV-infected adults, the CD4 count can be accurately estimated from the ALC using a baseline CD4 percent for at least 2 years after measurement.

## Background

The clinical value of routinely monitoring CD4 counts in HIV-infected adults with virologic suppression and immune reconstitution is questionable. After counts have risen to more than 300–350 cells/mm^3^, the proportion subsequently falling to less than 200 cells/mm^3^ is reported as only 1.1–2.9 % in retrospective studies [1–4]. The majority of these declines are predictable, transient, not directly due to HIV or anti-retroviral therapy (ART) and do not increase the risk of opportunistic infections. Reducing the frequency of CD4 testing has the potential for substantial cost savings - estimated at up to $18.1 million per year in the US [5]. Based on these findings, recent guidelines from the International AIDS Society (IAS) have recommended that among virologically suppressed individuals with CD4 counts above 500 cells/mm^3^ further monitoring of CD4 counts is optional [6]. Yet while patients may feel anxious over clinically insignificant fluctuations in the CD4 count, resistance to reduced monitoring has also been suggested.

We hypothesised that following virologic suppression and immune reconstitution, CD4 counts could be estimated using lymphocyte measurements from an automated full blood count (FBC) analyser. Such a method has not, to our knowledge, been reported before. FBCs are an important part of routine HIV care regardless of disease stage. In a retrospective cohort study, we aimed to derive and validate a simple heuristic algorithm that is able to estimate CD4 counts reliably.

## Methods

All available HIV viral loads (VL) and CD4 panels (including CD4/8 absolute values, percent and lymphocyte counts) were extracted from an electronic laboratory database. Where at least one CD4 or VL was available for a subject, absolute lymphocyte counts (ALCs) from FBC measurements were also extracted. Data was available from January 2008 to September 2013. CD4 panels were performed on a flow cytometer (BD Biosciences, New Jersey, USA), while FBC results were from an automated analyser (Beckman Coulter, California, USA). No patient information was available from this database.

Periods of virologic suppression were identified. Virologic suppression was defined as at least two consecutive viral loads less than 200 copies/ml within 400 days of each other, similar to other studies. During each period of virologic suppression, the first absolute CD4 measurement greater than 300 cells/mm^3^ and the accompanying CD4 percent was identified and used as baseline. For the following 720 days, CD4 and ALC measurements taken on the same day were collected. The baseline CD4 percent was used to estimate an absolute CD4 count from subsequent ALCs (CD4 estimate = ALC x baseline CD4 percent). Estimated CD4 values were compared with the actual value for accuracy (predicted CD4/actual CD4 x 100). Two factors were chosen as clinically relevant markers of accuracy:

1. Proportion of CD4 estimates within 10 % and 25 % of the actual value
2. Percentage deviation of accuracy from zero covering 90 % of estimates

Actual CD4 values less than 200 cells/mm^3^ were identified. Electronic and paper patient records were reviewed to determine if a cause could be identified – including serious illness requiring hospital admission within one month before CD4 measurement, cytotoxic chemotherapy, hepatitis C treatment or any other expected cause of lymphopenia.

A case–control study (1:2) of 150 individuals was then performed to identify factors predicting inaccurate results. Cases were defined as greater than 25 % underestimation of the actual CD4 value, while controls as estimates within 25 %.

Cases and controls were matched by expected confounders: baseline CD4 percent (exact) and days from baseline to CD4 estimate (within 28 days). Cases and controls were sampled randomly without replacement, each subject contributing only one estimate. Electronic and paper records were reviewed and compared for demographics, history of AIDS-defining infections, trough CD4 less than 200 cells/mm^3^, ART regimen and serious illnesses within one month prior to the date of CD4 or ALC testing.

Results from the case–control study were used to determine simple baseline clinical requirements which were predictive of accurate CD4 estimates. The algorithm was validated in a sample of 100 individuals by reviewing electronic and paper records. Where possible new CD4 estimates were generated when baseline clinical conditions were not met. Accuracy of estimates was compared between the two sets.

Statistical analysis was performed using R [7]. All samples were assumed to be independent (including for the case–control study), and p-values were interpreted with a two-tailed significance level of 5 %.

This study was performed at the Communicable Disease Centre (CDC), Tan Tock Seng Hospital Singapore. CDC is the main treatment centre for HIV in Singapore, with approximately 2,500 patients attending for HIV care annually. The study was approved by the National Healthcare Group Domain Specific Review Board, and access to the database approved by the Head of Infectious Diseases Department, Tan Tock Seng Hospital.

## Results

Fifteen thousand seventy-nine HIV VLs and 29,927 CD4 counts were available for analysis from 3,630 subjects. 3,885 paired CD4-ALCs covering 1,444 periods of virologic suppression in 1,388 subjects met the inclusion criteria for the study. Median frequency of VLs was 175 days (inter-quartile range (IQR): 127–245), median frequency of CD4 counts was 147 days (IQR: 105–178).

### Estimating CD4 values

Overall 83.3 % of CD4 estimates were within 25 % of the actual values, and 41.3 % within 10 %. 90 % of predicted CD4 counts were within 30 % of the actual value. Accuracy measures approximated the normal distribution with a mean 6.1 % underestimation. As a result, while 12.1 % CD4 counts were underestimated by more than 25 %, only 4.5 % were overestimated.

Lymphocyte counts correlated well between flow cytometry and automated FBC measurements (r = 0.970, p < 0.0001). A median absolute difference of 5.5 % was observed, but without significant variation of the mean from zero (one sample *t* test, *p* = 0.40). No significant difference between lymphocyte counts measured by CD4 panel or FBC were identified when results were stratified by absolute CD4 count or CD4 percent.

The ALC was only moderately correlated with the CD4 count (r = 0.589, p < 0.0001), and correlation with the CD4% was negative and substantially weaker (r = −0.216, p < 0.0001). Thus while the ALC is an accurate measure of lymphocyte counts, its ability to predict the CD4 count is limited.

**Fig. 1:**
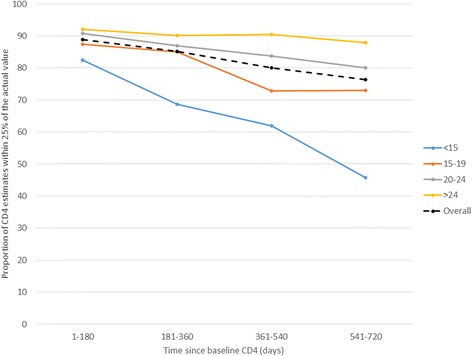
Accuracy of CD4 estimates as a function of time and stratified by baseline CD4 percent. ‘Overall’ includes all results

As expected, absolute CD4 count and CD4 percent were significantly higher with increasing time from baseline measurement (p < 0.0001). CD4 count was increasingly underestimated with time, and the degree of underestimation correlated with baseline CD4 percent (p < 0.0001). When the baseline CD4 percent was ≥25, more than 90 % of estimates were within 25 % of the actual value out to 720 days (Fig. 1).

### Case–control study

One hundred controls were matched to 50 cases. Case and controls were well matched by CD4 percent baseline and days (Table 1).

**Table 1:** Matched case–control study

	Case (*n =* 50)	Controls (*n =* 100)	Odds Ratio, p-value
Baseline CD4% (mean)	19	19	NS^a^
Days from baseline CD4% to estimated CD4 (mean)	393	393	NS^a^
Mean age (years)	45	47	NS^a^
Proportion >50 years	34 %	38 %	0.85 (0.46–1.55), NS^b^
Sex (male)	90 %	90 %	1 (0.35–2.84), NS^b^
History of AIDS defining illness or trough CD4 < 200	62 %	66 %	0.83 (0.44–1.57), NS^b^
First line ART	84 %	88 %	0.71 (0.28–1.73), NS^b^
Days ART (mean)	795	1004	NS^a^
<180 days	38 %	21 %	2.30 (1.09–4.87), 0.032^b^
<360 days	50 %	32 %	2.12 (1.06–4.26), 0.035^b^
<720 days	62 %	45 %	1.99 (1.00–3.99), 0.058^b^
ART switch (not due to virologic failure)	18 %	8 %	2.52 (0.91–7.01), 0.1 ^b^
Illness	16 %	0 %	0.0001 ^b^

^a^students *t* test, ^b^Chi square, Fischer’s exact

No significant differences in baseline lymphocyte counts, absolute CD4, CD8 or CD4/8 ratio were identified between cases and controls. There was also no significant difference between lymphocyte measurements by FBC and flow cytometry.

Controls had a significantly smaller *increase* in CD4 percent from baseline to estimate (p < 0.0001), and significantly lower CD4 counts (*p* = 0.002). Stable CD4 percent with time is clearly important for accurate results, and stability was not affected by age, gender, history of AIDS-defining illnesses or a documented CD4 trough of less than 200 cells/mm^3^. However, presence of serious illness requiring hospital admission predicted inaccurate estimates (*p* = 0.0001), as did less than a year since starting ART (*p* = 0.035). Change of ART in virologically suppressed hosts was common and more frequent among cases, but this rate were not statistically significantly different from controls. 15/17 (88.2 %) of switches were between nucleoside reverse transcriptase inhibitors, primarily from stavudine to zidovudine or tenofovir.

### Heuristic algorithm and validation

The following baseline parameters for CD4 estimates were chosen for the validation phase of the study:

1. Baseline CD4 percent ≥20, Absolute CD4 ≥ 300
2. Virologic suppression (<200 copies/ml)
3. More than one year since starting ART or a change in regimen
4. No serious concomitant illness requiring hospital admission or expected to cause lymphopenia

A new sample of 100 individuals was screened following the above algorithm, yielding 292 CD4 estimates. 5 subjects were excluded from validation as no results where available meeting baseline criteria, while 49 subjects required recalculation of estimates. Primary reasons for recalculation were ART related – treatment for less than one year in 28 (51.9 %) and switch of therapy in 22 (40.7 %). 243 CD4 estimates were analysed after validation. There was no statistically significant difference in the average time since baseline CD4% in either group (derivation 352 days vs. validation 359 days), while mean baseline CD4 percent increased marginally from 25.4 to 26.8 % (*p* = 0.002, *t*-test).

The algorithm significantly improved accuracy of CD4 estimates. The proportion of estimates within 25 % of the actual value increased from 86.0 to 93.4 % (*p* = 0.007), and within 10 % from 46.6 to 57.2 % (*p* = 0.015). 90 % confidence intervals for estimates narrowed from 28 to 22 %.

Estimates were significantly more accurate in the first versus second year for the derivation cohort (mean 1.8 % vs. 6.2 % underestimation, *p* = 0.044). This narrowed after validation (mean 0 % v 4.7 % underestimation, *p* = 0.002).

### CD4 dips to less than 200 cell/mm^3^

From the initial cohort, 49 (1.3 %) CD4 counts from 41 subjects were less than 200 cells/mm^3^. 34 (69.3 %) were identified in the first year of care. 31/49 of these were also predicted by the algorithm (sensitivity 63.3 %, 95 % CI: 48.3–76.6; specificity 98.8 %, 95 % CI 98.4–99.1). After reviewing the charts of the 41 subjects, serious illnesses were identified in 10. CD4 dips were typically transient, in only 6 of 39 subjects (15.4 %) with follow up CD4 counts were dips below 200 cells/mm^3^ sustained for more than one CD4 measurement. No AIDS-defining opportunistic infections were diagnosed when the CD4 count was less than 200 cells/mm^3^.

## Discussion

In immune-reconstituted, virologically suppressed and clinically stable HIV-infected adults, the CD4 count can be accurately estimated by multiplying the absolute lymphocyte count by a baseline CD4 percent. This estimate is within 25 % of the actual value almost 95 % of the time for at least the first two years from baseline measurement.

Previous studies have demonstrated that using ALC cut-offs to predict an absolute CD4 count of <200 cells/mm^3^ or response to ART is unreliable for clinical use [7, 8]. The primary reason for this lack of concordance between ALC and CD4 is variability in the CD4 percent rather than large discrepancies between lymphocyte counts when measured by an automated FBC analyser or lymphocyte subset flow cytometer. Stability of the CD4 percent is crucial for accurate CD4 estimates using the method outlined here. The CD4 percent has less physiological variability than the absolute count, and following immune reconstitution increases in the CD4 percentage are proportionally smaller [9].

Data from this study supports reducing the frequency of CD4 monitoring in clinically stable, virologically-suppressed and immune-reconstituted adults. Similar to previous studies, the proportion of CD4 values less than 200 cells/mm^3^ was low – only 1.3 % and was generally predictable, transient and not associated with opportunistic infections. The clinical utility of CD4 counts in this population is limited, yet, measurement continues. We propose that where patients or healthcare providers prefer knowing a CD4 value, this study provides a simple and accurate method of estimating it. The reassurance of this estimate may facilitate implementation of recent guidelines to reduce the frequency of testing and achieve anticipated cost savings. At our institution, CD4 panels are substantially more expensive than FBC counts (113.42 vs 25.74 SGD, before government subsidies). For the 100 patients in the validation phase of this study, stopping CD4 counts was equivalent to a cost saving of $27,561 over two years.

This study has several limitations. It is retrospective and was performed in a population that was largely male with a low incidence of intravenous drug use and hepatitis C infection (2 %) [10]. Zidovudine or stavudine-based anti-retroviral therapy were standard first line treatments until recently, and the association of these agents with lymphopenia may reduce the accuracy of this method. A VL periodicity of 400 days was chosen to reflect local clinical practice, where annual testing is sometimes necessary due to cost considerations. Inevitably this does not guarantee continuous virologic suppression. Treatment interruptions which were unrecognised or not confirmed virologically were not excluded from this analysis. The number of CD4 estimates per individual was not restricted. However, more frequent monitoring is more likely during periods of clinical instability or early during the treatment course. Both limitations are likely to bias this study towards inaccurate CD4 estimates and are mitigated by the validation phase.

An estimated CD4 count within 25 % of the actual value was chosen *a priori* as clinically adequate. What degree of accuracy is acceptable is debatable, but this level is similar to expected physiological variability [9, 11]. Finally, a single set of simple clinical rules was chosen for validation in a relatively small cohort which may not have been representative. These rules may not be the best possible for accurate CD4 estimates.

## Conclusion

In virologically-suppressed and immune-reconstituted HIV-infected adults, the CD4 count can be accurately estimated from the ALC using a baseline CD4 percent for at least 2 years after measurement.
